# Effectiveness of a modified group cognitive behavioral therapy program for anxiety in children with ASD delivered in a community context

**DOI:** 10.1186/s13229-020-00341-6

**Published:** 2020-05-13

**Authors:** Abbie Solish, Nora Klemencic, Anne Ritzema, Vicki Nolan, Martha Pilkington, Evdokia Anagnostou, Jessica Brian

**Affiliations:** 1 Holland Bloorview Kids Rehabilitation Hospital, Toronto, ON Canada; 2 Autism Research Centre, Bloorview Research Institute, Toronto, ON Canada; 3 Child Development Institute, Toronto, ON Canada; 4 Lighthouse Child & Adolescent Psychology, Ottawa, ON Canada; 5 Department of Paediatrics, University of Toronto, Toronto, ON Canada

**Keywords:** Autism, Autism spectrum disorder, Anxiety, Intervention, Cognitive behavioral therapy, Group, Implementation, Community

## Abstract

**Background:**

Youth with autism spectrum disorder (ASD) experience high rates (approximately 50–79%) of comorbid anxiety problems. Given the significant interference and distress that excessive anxiety can cause, evidence-based intervention is necessary in order to reduce long-term negative effects. Cognitive behavioral therapy (CBT) has demonstrated efficacy for treating anxiety disorders across the lifespan, both in individual and group formats. Recently, modified CBT programs for youth with ASD have been developed, showing positive outcomes. To date, these modified CBT programs have primarily been evaluated in controlled research settings.

**Methods:**

The current community effectiveness study investigated the effectiveness of a modified group CBT program (Facing Your Fears) delivered in a tertiary care hospital and across six community-based agencies providing services for youth with ASD. Data were collected over six years (*N* = 105 youth with ASD; ages 6–15 years).

**Results:**

Hospital and community samples did not differ significantly, except in terms of age (hospital *M* = 10.08 years; community *M* = 10.87 years). Results indicated significant improvements in anxiety levels from baseline to post-treatment across measures, with medium effect sizes. An attempt to uncover individual characteristics that predict response to treatment was unsuccessful.

**Conclusions:**

Overall, this study demonstrated that community implementation of a modified group CBT program for youth with ASD is feasible and effective for treating elevated anxiety.

## Key points

- Youth with autism spectrum disorder (ASD) experience high rates of anxiety
- Modified cognitive behavioral therapy (CBT) programs for youth with ASD are efficacious based on well controlled lab-based studies
- We examined the effectiveness of community implementation of a modified CBT program (Facing Your Fears) in a large sample of youth with ASD, aged 6 to 15 years by comparing results from a tertiary hospital program versus community program delivery
- Hospital and community-based program delivery resulted in comparable significant improvements in anxiety following treatment
- An attempt to uncover individual predictors of treatment response was unsuccessful
- Findings demonstrate that community implementation of a modified group CBT program for youth with ASD is feasible and effective for treating elevated anxiety

## Introduction

Autism spectrum disorder (ASD) is a complex neurodevelopmental disorder characterized by impairments in social interaction and communication, and the presence of stereotyped, repetitive behavior, and/or restricted interests [1]. Based on recent reports, ASD affects as many as 1 in 59 children [2]. Children and youth with ASD experience high levels of anxiety [3]. A recent review of studies examining the prevalence of anxiety disorders in ASD, based on International Statistical Classification of Diseases (ICD), or Diagnostic and Statistical Manual of Mental Disorders (DSM) criteria, revealed high rates of comorbidity, with the majority reporting a prevalence of around 50%, although some reported rates as high as 78–79% [4–6]. Excessive anxiety causes considerable distress and interference with daily functioning [7], and without intervention, may become more impairing with age, thereby negatively impacting long-term prognosis and outcome [8]. Addressing anxiety is an important treatment goal for children and youth with ASD, in order to mitigate the long-term cycle of maladaptation.

Studies examining the use of psychosocial interventions such as cognitive behavioral therapy (CBT) to treat anxiety in ASD have emerged over the past decade [9]. A small number of CBT programs have been designed or adapted specifically for children with ASD, yielding improvements in anxiety symptoms in response to both group and individual formats [10–15]. An initial systematic review and meta-analysis [16], and a more recent review of randomized control trials (RCTs) [17] converge to conclude that in general, psychosocial interventions are superior to waitlist control or treatment as usual (TAU) conditions. The majority of studies report significant reductions in anxiety symptoms in children receiving CBT, based on self-, parent-, and/or clinician-report, or by children no longer fulfilling criteria for an anxiety disorder [10, 11], with one outlier [18] failing to find an advantage for CBT when compared to a Social Recreational program. Taken together, there is evidence supporting favorable reductions in anxiety for youth with ASD and anxiety in response to CBT-based interventions, but evidence gaps remain. Notably, most evidence comes from efficacy studies that rely heavily on involvement from the program developers or on carefully controlled research-based delivery—it remains to be demonstrated whether such programs are feasible and effective in wide-scale community implementation [19].

Establishing the transportability of efficacious interventions into community settings is a necessary step in increasing access. Despite the growing body of evidence for the efficacy of CBT for youth with ASD, there remains a gap between research-based and community implementation of these interventions [20]. Recently, program developers demonstrated the initial portability of the Facing Your Fears program in partnership with an outpatient service at a Pediatric Hospital in Nova Scotia, Canada (*n* = 16) [21]. Over half of group participants saw meaningful reductions in anxiety thus demonstrating, in a small sample, the promise of community implementation. Evaluation of effectiveness through a large-scale community trial remains to be conducted and is the necessary next step in establishing an evidence base for such programs that can inform practice and policy.

Questions also remain about individual differences in treatment response, with possible influences of anxiety and ASD symptoms, cognitive functioning, or language ability. Some evidence points to higher anxiety in children with milder ASD symptoms [22–24] (but findings are inconsistent [25, 26]), or in those who are cognitively higher functioning [22, 27] (also with mixed findings [3, 28]). The role of age is also unclear, with older children experiencing higher anxiety in some studies [29, 30], but not others [27, 31]. The impact of these individual differences on treatment response may inform future research, programming, or policy decisions.

The current community effectiveness study examined the *effectiveness* of a manualized group CBT program (Facing Your Fears; FYF [32]) by examining outcomes for participants receiving treatment in a tertiary hospital and community-based ASD service settings. This study aimed to answer three questions:

1. Is modified group CBT for children and youth with ASD effective when implemented as a clinical service?
2. Is the FYF program less effective in community implementation than when delivered in a specialized hospital setting?
3. Do individual child characteristics significantly predict treatment response?

## Method

### Participants

One hundred and seventeen children/youth aged 6.91 to 15.33 years enrolled in a 14-week CBT clinical intervention adapted for individuals with ASD (FYF [32]) either in a specialized hospital setting (main site) or through one of six community-based agencies providing services for youth with ASD (community sites). Eligibility required an ASD diagnosis, broadly average intellectual ability, elevated levels of anxiety, and no significant behavioral disturbances that preclude group participation. Of the 117 children enrolled, 12 did not complete group due to disruptive behavior/ not being able to manage the group context (*n* = 6), missing > 3 sessions (*n* = 5), or change in family availability (*n* = 1). A total of 105 children were included in the analyses (76 males, 29 females; see Table 1 for sample characteristics). This study received approval as a retrospective chart review by the research ethics board at the main hospital.

**Table 1:** Participant baseline characteristics

Variable	*n*	Mean (SD)	Range
Age (years)	105	10.47 (1.75)	6.91–15.33
IQ	74	103.70 (14.54)	69–135
ASD symptoms			
SRS Total T-score	104	74.90 (9.79)	49–90
SCQ Total score	105	16.52 (5.94)	2–30
Anxiety (total scores)			
Parent-report SCAS	104	34.88 (13.29)	10–90
Child-report SCAS	104	29.76 (13.52)	4–71
Parent-report SCARED	102	32.81 (11.41)	6–72
Child-report SCARED	99	24.57 (12.21)	0–67
Behavior (BASC-2)			
Externalizing	103	58.62 (11.46)	40–102
Internalizing	102	65.34 (12.69)	38–105
Behavioral Symptoms Index (BSI)	102	70.32 (10.15)	52–107

*Note*. *IQ* Full-Scale IQ (FSIQ) or Generalized Ability Index (GAI) from WASI, WASI-II, WISC-IV, WISC-V, WPPSI-III, or Stanford-Binet Intelligence Scales, Fifth Edition (SB5). *SRS* Social Responsiveness Scale, First and Second Editions, *SCQ* Social Communication Questionnaire, *SCAS* Spence Children’s Anxiety Scale (Total Score), *SCARED* Screen for Child Anxiety Related Emotional Disorders (Total Score), *BASC-2* Behavior Assessment System for Children, Second Edition (*T* scores)

All children had a diagnosis of ASD (DSM-5 or DSM-IV Autism, Asperger Syndrome, or Pervasive Developmental Disorder, Not Otherwise Specified) made by a physician or psychologist prior to enrollment in the program. Average intellectual functioning (full scale IQ > 80) was confirmed for most participants (*n* = 64) during screening or based on formal reports (if within two years of group). A small number (*n* = 4) had below-average intellectual functioning, due to enrollment prior to assessment. Anxiety symptoms were assessed through semi-structured telephone interviews with parents, and parent- and child-reported questionnaires. Comorbid difficulties (e.g., depressed mood, attention-deficit/hyperactivity disorder, learning disability) did not exclude children who were considered likely to be able to manage the group context.

### Procedure

Eligibility screening was conducted, within 2 months prior to group participation, by registered clinical doctoral-level psychologists (or those in the process of registering), or pre-doctoral-level psychology interns. A structured telephone interview (45–60 min) covered previous assessments and diagnoses, educational history, prior experience in group settings, and detailed information about past and current mood and anxiety symptoms and management (formal diagnoses, specific fears, phobias, avoided situations, medications, previous interventions). Next, parents and children attended individual in-person screenings for cognitive assessment and child- and parent-baseline questionnaires. Additional pre-intervention data were collected during the first treatment session (measures described below).

The group intervention was run according to the *Facing Your Fears Facilitator’s Manual* [32]. Groups at the main site were run primarily by psychologists and psychology interns who had participated in training workshops by the program developers (Judy Reaven [JR] and Audrey Blakeley-Smith [ABS]) and/or who had undergone video-based fidelity monitoring with JR. To attain fidelity, sessions were video-taped and sent to program developer, JR. She watched them and scored them based on her fidelity checklist developed for the FYF program. Following her review of every 2–3 sessions, the team met with JR over the telephone to discuss their use of intervention procedures. All staff at the main site attained > 80% fidelity in implementing the program for each session. Every time a new community partner was added, the same fidelity process was repeated, and all community sites received > 80% fidelity on their first group delivery; the last community site was added in year 5 of the 6-year project, allowing for fidelity checks throughout much of the length of the project. All groups at the main and community sites were run with at least one clinician who had already achieved 80% fidelity. In total, seven psychologists and nine psychology interns at the main site were trained; group leaders also included three social workers, two psychology practicum students, one developmental pediatrician, and one behavior therapist, all of whom were trained by lead psychologists or program developers. Community sites sent at least one staff member to the main site to co-lead FYF prior to facilitating groups at their own agencies. Group leaders across six community sites included 28 behavior therapists, 7 social workers, 2 psychologists, 1 psychology intern, 1 practicum student, 2 family counsellors, and 1 early childhood educator. In all but one case, one main site lead psychologist co-led each group in the community. Ongoing consultation phone calls with JR took place after fidelity monitoring was complete, allowing group leaders to consult regularly with an expert while running the groups.

Twenty-six groups (12 main sites; 14 community) of 3 to 5 child/parent dyads each, took place over a six-year period. Groups involved 14 weekly 1.5-h sessions (12–13 sessions for four community groups due to staffing constraints). Per FYF protocol, initial sessions focused on emotion regulation (e.g., identifying emotions, gaining awareness of anxiety-related physical symptoms and thought patterns, practicing relaxation, using “helpful thoughts”), while later sessions focused on hierarchical exposure practice (i.e., facing fears). Child participants and at least one parent spent time working together and apart in separate parent and child groups. Weekly home practice was assigned and reinforcement strategies were used to encourage exposure practice between sessions.

### Measures

Wechsler Abbreviated Scale of Intelligence (WASI and WASI-II) [33, 34]. The WASI or WASI-II was used at baseline to estimate full-scale IQ using two verbal subscales and two nonverbal subscales. Both editions of the WASI demonstrate good internal consistency (split-half reliability coefficients > .80 within child sample for all subtests), good test-retest stability, and good internal validity. The WASI and WASI-II are highly correlated (*r* range = .71 to .88 across subtests).

Social Responsiveness Scale (SRS and SRS-2) [35, 36]. The SRS or SRS-2 was used at baseline to characterize participants’ ASD symptoms. This 65-item caregiver-report questionnaire for children aged 4–18 consists of five sub-scales: Social Awareness, Social Cognition, Social Communication, Social Motivation, and Autistic Mannerisms; and a Total T score. Items are rated from 0 (“not true”) to 3 (“always true”), with T-scores > 60 within the clinical range. The SRS and SRS-2 have well-documented reliability (e.g., strong internal consistency, α typically > .90) and validity. We used both versions because version 2 was released while data collection was ongoing; the SRS and SRS-2 have identical content [36].

Social Communication Questionnaire (SCQ) [37]. The SCQ, also used at baseline, is a 40-item parent-completed questionnaire based on the Autism Diagnostic Interview -Revised (ADI-R) [38]. The SCQ has good internal consistency (α ranging from .84 to .93), and validity (i.e., most items discriminate between ASD and non-ASD).

Spence Children’s Anxiety Scale, Child and Parent versions (SCAS) [39]. The SCAS was used at screening and post-intervention. This measure of anxious symptoms (44 items on the child self-report, and 38 items on the parent-report) has good full-scale internal consistency (α > .90) and acceptable test-retest reliability after a 6-month delay (*r* = .60) [39]. Internal consistency estimates for the current sample ranged from .85 to .90.

Screen for Child Anxiety Related Emotional Disorders, Child and Parent versions (SCARED) [40]. The SCARED is a 38-item measure of anxious symptoms, with both child and parent versions (both used pre- and post-intervention). The SCARED has strong full-scale internal consistency (α = .93), good test-retest reliability (*r* = .86), and good discriminant validity in a child clinical sample [40]. Internal consistency estimates for the current sample ranged from .86 to .92.

Behavior Assessment System for Children, Second Edition (BASC-2) [41]. The BASC-2 Parent Rating Scales were used in the current study, both pre- and post-intervention; this includes 150 (adolescent) or 160 (child) behavioral items rated based on frequency (*never* to *almost always*). Three age-based standardized composite scores were used: *externalizing problems* (e.g., disruptive behavior, aggression, oppositionality), *internalizing problems* (e.g., mood and anxiety difficulties, stress-related physical symptoms), and *behavioral symptoms Index* (BSI; e.g., atypical behavior, social concerns, and attentional problems). The BASC-2 has good internal consistency for individual (> .80) and composite (> .90) scales, and good test-retest reliability for individual (70-80%) and composite (> 80%) scales, across age groups.

Group Questionnaires, Parent and Child versions. Developed by authors (AS and JB) to be used pre- and post-intervention. Includes quantitative ratings (e.g., levels of anxiety and its interference), and qualitative responses (e.g., strategies a parent is currently using). Item development was informed by knowledge of anxiety and ASD and on parenting children with ASD. See Appendix A for detailed description of items and domains used in analyses.

Satisfaction Questionnaire. Parents’ satisfaction with the program was measured following intervention. The questionnaire includes four qualitative responses (e.g., what parents liked most and least about the program) and 18 quantitative ratings (e.g., quality of the program, amount and type of help received, efficacy, quality of instruction) rated on a 1-9 scale, thus yielding a score out of 162.

## Results

### Baseline group comparisons

One-way ANOVAs compared baseline characteristics of participants at the main site (*n* = 51) and community-based settings (community; *n* = 54). Groups differed only by age (Table 2). ANCOVA, controlling for age, showed no significant differences between groups on pre-treatment measures of anxiety or other behavioral difficulties (Table 3).

**Table 2:** ANOVAs comparing groups on baseline characteristics

Variable	Main site mean (*SD*)	Community sites mean (*SD*)	*F* (df)	*p*
Gender	74.1% male	70.6 % male	.16 (1, 103)	.69
Age	10.08 (1.71)	10.87 (1.72)	5.60 (1, 103)	.02
IQ	102.68 (15.00)	104.78 (14.17)	.38 (1, 72)	.54
SRS (total *t* score)	73.79 (10.36)	76.06 (9.12)	1.40 (1, 102)	.24
SCQ (total score)	16.77 (6.08)	16.26 (5.83)	.19 (1, 103)	.67

*Note*. *SRS* Social Responsiveness Scale, First and Second Editions, *SCQ* Social Communication Questionnaire

**Table 3:** Baseline behavioral measures across groups

Measures	Main site mean (SD)^a^	Community sites mean (SD)^a^	*F* (df)^b^	*p*
Parent report				
SCARED	31.89 (9.96)	33.84 (12.88)	.49 (1,99)	.48
SCAS	34.47 (13.01)	35.32 (13.69)	.42 (1,101)	.52
BASC-2 (*t* scores)				
Externalizing	57.55 (11.74)	59.76 (11.15)	1.76 (1, 100)	.19
Internalizing	64.11 (11.40)	66.67 (13.95)	.83 (1,99)	.37
BSI	69.85 (11.51)	70.84 (8.51)	.50 (1,99)	.48
Parent questionnaire				
Effective	3.45 (1.20)	3.72 (1.18)	1.06 (1, 97)	.31
Avoidance	2.70 (1.33)	2.82 (1.17)	.07 (1,95)	.80
Interference	5.00 (1.50)	5.27 (1.41)	.78 (1,97)	.38
Family impact	5.17 (1.84)	5.28 (1.79)	.01 (1, 97)	.92
Child report				
SCARED	23.10 (11.21)	26.20 (13.15)	1.12 (1,96)	.29
SCAS	28.96 (12.18)	30.59 (14.86)	.51 (1,101)	.48
Child questionnaire				
Amount of worry	3.34 (2.04)	3.83 (2.07)	.83 (1, 96)	.37
Interference/distress	3.22 (2.48)	3.88 (2.40)	1.40 (1,96)	.24

*Note*. *SCARED* Screen for Child Anxiety Related Emotional Disorders, total score, *SCAS* Spence Children’s Anxiety Scale, total score, *BASC-2* Behavior Assessment System for Children, Second Edition; see Appendix A for information on Parent and Child Questionnaires

^a^Unadjusted means reported

^b^Adjusted *F*-statistic from ANCOVA controlling for age

### Treatment effects

#### Combined sample

Paired samples *t* tests revealed significant improvements from pre- to post-treatment on 11 (of 13 tested) measures of anxiety and behavior (see Table 4). Family-wise error corrections were made for analyses that used measures with multiple domains; specifically, adjusted *p*’s < .02 (BASC-2), .01 (Parent Questionnaire), and .03 (Child Questionnaire). All other tests (i.e., SCAS and SCARED) used critical *p* < .05.

**Table 4:** Pre- and post-treatment performance on behavioral measures (combined sample)

Measures	Pre-treatment mean (SD)	Post-treatment mean (SD)	*T* (df)	*P*^a^	Effect size^b^
Parent report					
SCARED	33.28 (11.21)	26.00 (10.45)	8.00 (97)	< .001	.67
SCAS	35.23 (13.35)	28.68 (11.71)	6.10 (99)	< .001	.52
BASC-2 (*T* scores)					
Externalizing	59.13 (12.04)	56.19 (11.01)	3.98 (85)	< .001	.24
Internalizing	65.36 (12.23)	59.26 (11.63)	6.20 (87)	< .001	.51
BSI	70.31 (10.19)	66.13 (10.03)	5.98 (87)	< .001	.41
Parent questionnaire					
Effective	3.59 (1.19)	5.02 (1.33)	-8.82 (92)	< .001	− 1.14
Avoidance	2.79 (1.24)	2.20 (1.20)	3.98 (89)	< .001	.48
Interference	5.13 (1.46)	4.28 (1.45)	5.71 (92)	< .001	.59
Family impact	5.27 (1.81)	4.28 (1.91)	4.66 (92)	< .001	.53
Child report					
SCARED	24.84 (12.18)	20.17 (13.35)	4.42 (96)	< .001	.36
SCAS	29.83 (13.56)	24.52 (15.17)	3.77 (102)	< .001	.37
Child questionnaire					
Amount of worry	3.60 (2.06)	3.49 (1.89)	.55 (97)	.58	.05
Interference/distress	3.56 (2.45)	3.29 (2.13)	1.19 (97)	.24	.12

*Note*. *SCARED* Screen for Child Anxiety Related Emotional Disorders, total score, *SCAS* Spence Children’s Anxiety Scale, total score, *BASC-2* Behavior Assessment System for Children, Second Edition

^a^Significance based on corrected *p* values

^b^Effect size used modified Cohen’s *D* to correct for correlation between dependent variables [*d* = *t*(2(1-r)/n)^1/2^] [48]

#### Within-site effects

*t* tests for the main and community sites separately yielded the same significant intervention effects (as combined) with the exception of one: Child-reported anxiety (SCAS) did not change significantly in the community group (*t* (49) = 1.62, *p* = .11; pre-treatment *M* = 30.76, SD = 14.96; post-treatment *M* = 26.92, SD = 17.29), whereas it did for the main site (*t* (52) = 4.23, *p* < .001; pre-treatment *M* = 28.96, SD = 12.18; post-treatment *M* = 22.25, SD = 12.61).

### *Cross-site comparisons*

Change scores were calculated for the 11 variables identified as changing significantly over time. Negative change scores represent improvement in symptoms except for the Parent Questionnaire variable “effective” (Table 5). One-way ANCOVAS, controlling for age, revealed no group differences in any of the change scores, and negligible effect sizes.

**Table 5:** Change scores for combined sample and for main and community samples separately

Variable	Change score mean (SD) Combined sample	Change score mean (SD) Main site	Change score mean (SD) Community sites	*p*^a^	Effect size^b^
Parent report					
SCARED	− 7.28 (9.01)	− 7.14 (8.00)	− 7.43 (10.08)	.79	.001
SCAS	− 6.54 (10.72)	− 6.30 (9.46)	− 6.79 (11.99)	.66	.002
BASC-2 (*T* scores)					
Externalizing	− 2.94 (6.86)	− 2.98 (7.04)	− 2.90 (6.74)	.83	.001
Internalizing	− 6.10 (9.23)	− 6.09 (8.40)	− 6.11 (10.14)	.82	.001
BSI	− 4.18 (6.56)	− 4.47 (7.11)	− 3.88 (5.99)	.84	< .001
Parent questionnaire					
Effective	1.43 (1.57)	− 1.61 (1.49)	− 1.23 (1.64)	.39	.008
Avoidance	− .59 (1.40)	.55 (1.37)	.63 (1.46)	.80	.001
Interference	− .85 (1.44)	.83 (1.53)	.88 (1.35)	.83	< .001
Family impact	− .98 (2.04)	1.13 (2.30)	.82 (1.70)	.41	.008
Child report					
SCARED	− 4.67 (10.42)	− 6.13 (12.08)	− 3.06 (8.03)	.15	.022
SCAS	− 5.31 (14.30)	− 6.70 (11.53)	− 3.84 (16.75)	.29	.011

*Note*. *SCARED* Screen for Child Anxiety Related Emotional Disorders, total score, *SCAS* Spence Children’s Anxiety Scale, total score, *BASC-2* Behavior Assessment System for Children, Second Edition

^a^Significance based on between-site ANCOVA (controlling for age)

^b^Effect size is Cohen’s *D*

### Predictors of treatment response

Data were combined across sites (*N =* 105) to examine correlations between anxiety change scores and four participant baseline characteristics (age, Full-Scale IQ, Total ASD symptoms on SRS and SCQ). Using Pearson’s bivariate correlations, only one correlation reached significance, with parent-rated improvement in anxiety (SCARED) being negatively correlated with child’s IQ, *r* = − .32, *p* = .008. Four separate one-way ANOVAs, with gender as the grouping variable, revealed no differences with respect to change scores on any of the pre-post anxiety measures (all *p*’s > .25).

Change scores for the main anxiety measures (SCAS and SCARED, parent- and child-report) were also examined as dependent variables in four linear regression models using the five participant characteristics as predictors with critical *p* set to < .01 (.05/4). No models reached statistical significance. IQ approached significance as a negative predictor of the change in parent-report SCARED score, *β* = .29, *p* = .022.

### Retention and parent satisfaction

Out of a total 117 children enrolled, 12 were unable to complete the intervention protocol with fewer than 3 missed sessions, yielding a retention rate of 89.74%.

Parent-rated satisfaction was high, with a mean of 140.62 (SD = 15.43) out of a possible 162. Correlations revealed a general pattern of positive associations between satisfaction and improvements in anxiety symptoms, with moderately strong correlations between parent-reported anxiety (on the SCAS) and the following: How (improved) is your child’s anxiety at this point as compared to the start of the group? (*r* = − .34, *p* = .001; the only association to retain significance when using family-wise error correction (*p* < .0027); The content of information presented was (how helpful)? (*r* = .30, *p* = .003); in-session exposures with your child during group sessions were (how helpful)? (*r* = .30, *p* = .004); and how confident do you feel managing your child’s feelings of anxiety now? (*r* = .29, *p* = .005).

## Discussion

As hypothesized, youth with ASD and anxiety experienced an overall reduction in anxiety symptoms following a group CBT program tailored to this population. Improvements were similar across hospital and community settings, and retention and parent satisfaction were high. These findings demonstrate the feasibility, acceptability, and effectiveness of the FYF program when implemented in community settings with adequate up-front training and ongoing support.

Parent-reported improvements in anxious symptoms yielded mainly medium-sized effects, in line with previous studies in well-controlled settings [10–12, 42], with reported reductions in anxiety symptoms very similar to those previously reported using the same measure (SCARED) [11]. Interestingly, the magnitude of change in total scores was almost identical for the two instruments used in the current study, and the effect sizes were comparable. As such, we are not able to recommend one instrument over the other for future applications. Our smaller effects on externalizing difficulties are not surprising, given that disruptive behavior was not a direct intervention target. Moreover, youth were excluded if they displayed significant disruptive behaviors, leaving less room for reduction (i.e., a possible floor effect). Indeed, mean externalizing behavior ratings did not reach clinically significant levels before or after treatment. In contrast, mean internalizing behavior ratings (which include anxious behavior) dropped from above to below the clinical cut-off on the BASC-2.

A large effect was seen for parents’ report of their own increased levels of confidence and perceived effectiveness in parenting their anxious children. This likely reflects parents’ high levels of involvement in the intervention, which includes specific training and practice with management of children’s anxious symptoms. Parents’ experienced gains have the potential for long-term impact.

Children’s self-reported changes in anxiety symptoms yielded smaller effects than parents’ reports, with only some measures indicating small to medium effects (SCAS, SCARED). Positive effects emerged from well-established measures which were likely psychometrically stronger than the questionnaire developed for this study, which detected no change. Determining which (if any) self-report measures are valid for accessing emotional states of youth with ASD and anxiety has been a longstanding concern in this field [12, 14]. In some studies, parent and child SCAS anxiety ratings improve in concert [10] or are highly correlated (e.g., *r* = .66 at baseline) [42], suggesting that children/youth with ASD do have reliable awareness of their own anxiety symptoms. In contrast, however, Sofronoff and colleagues [12] found that children did not provide valid data when completing the SCAS. In another study using the Multidimensional Anxiety Scale for Children (MASC), child self-report indicated improvements in anxious symptoms over time even in the absence of intervention, while parent report suggested improvements in symptoms only for children who received intervention [14]. In the current study, some individuals appeared to have reduced insight into their own struggles (e.g., rating themselves as experiencing low levels of anxiety, despite parent-reported interference in daily life), but it remains possible that individuals attribute these daily struggles to factors other than anxiety; the individual lived experience cannot be ascertained from the current study design. Notably, a vast majority of children did acknowledge at least one or two significant fears, and correlations between child and parent report on both SCAS and SCARED baseline measures were moderate (*r* = .44 and *r* = .39, respectively), suggesting that children had some awareness of and ability to report their own anxious symptoms.

Our attempts to identify individual characteristics that predict response to treatment were unsuccessful. Baseline IQ, age, and ASD symptomology did not predict levels of change in anxious symptoms over time, nor did the child’s gender, but we acknowledge that this might be due to constraints of the present sample (e.g., most participants with IQ > 80). One puzzling correlation suggested that higher IQ was associated with smaller improvements in (parent-reported) anxiety. Given that this association was not seen with other measures of anxiety, it may be a finding particular to this sample, and requires further inquiry.

### Strengths and limitations

Strengths of this study include its focus on implementation in a community setting. Interdisciplinary community-based providers, with supervision from mental health professionals, learned to deliver FYF, achieved implementation fidelity, and helped children experience significant reductions in anxiety.

Our sample was large and diverse in terms of age, IQ, ASD, and anxiety symptoms, and similarities between groups at baseline allowed for robust comparisons between groups. The retention rate across settings was very high (i.e., 90% of those who enrolled were able to complete the program with fewer than three missed appointments), demonstrating feasibility and acceptability, and revealing that our results are not exclusively based on a select sub-group able to complete the 14 weeks; this supports the generalizability of our findings and sustainability of the program.

Our participants were comparable to those included in previous efficacy studies [e.g., 11, 21] with respect to baseline anxiety symptoms (e.g., [11] reported baseline SCARED scores of 33 and 27 for parent and child-report, respectively) and Full-Scale IQ (i.e., group mean within average limits, ranging from approximately 70 to 130), but it is difficult to determine whether ASD symptoms were comparable across studies, as most other studies describe their sample with respect to diagnostic categories rather than based on a measure of ASD symptoms. A strength of the current study is our use of the SCQ and SRS as continuous measures of ASD symptoms, with considerable range across individuals. In terms of ASD symptomatology, we do acknowledge that a small number of cases had SCQ scores below the cut-off of > 11 that has been recommended in the literature [43], but note that 83% of the sample exceeded this cut-off. It is not uncommon for a single measure to fail to capture all the relevant symptoms in an individual with ASD (and indeed the recommended best practice involves combining information from multiple sources and using that information to guide clinical judgment [44]), so these values are not unexpected. Moreover, in more mildly affected children, it is not uncommon for symptoms to be relatively undetected in the 4- to 5-year age range [45], which is the reference point for the majority of items on the SCQ.

Limitations include the lack of control group. However, our focus was on establishing the “real-world” effectiveness of the program, rather than its efficacy which has already been demonstrated through tightly controlled RCT studies. Moreover, confidence in our findings is supported by the consistency in outcomes across studies. We also had limited demographic information about factors (e.g., socio-economic and cultural factors) that may influence response to treatment. Given that the programs were run within the context of usual clinical care, we were not able to attain individual-level information about families’ socio-demographic characteristics. However, all of the intervention groups (both main and community sites) took place in Toronto, Ontario, which has been identified as one of the most culturally diverse cities in the world, with 200 different ethnic groups and over 50% of its population born outside of Canada [46]. Given that FYF was delivered as part of a clinical, rather than research, program it was also not possible to exclude children participating in concurrent treatments (e.g., medications, other behavioral programs). Finally, we collected data only from parents and children themselves, and blinding to treatment was not possible; thus, we did not have independent measures of outcomes. In future, therapists’ ratings of observed changes or more objective ratings from teachers blinded to treatment condition would strengthen the evidence.

Findings demonstrate that community implementation of a modified group CBT program for youth with ASD is feasible and effective for treating elevated anxiety. This evidence supports the provision of modified group CBT for youth with ASD within appropriately resourced community settings which has the potential to substantially increase access to mental health supports for individuals with impairing anxiety symptoms.

## Conclusions

Youth with autism spectrum disorder (ASD) experience high rates of anxiety. Although modified cognitive behavioral therapy (CBT) programs for youth with ASD have been shown to be efficacious based on well controlled lab-based studies, evidence of *effectiveness* within the context of community implementation is lacking. This study examined the effectiveness of community implementation of a modified CBT program (Facing Your Fears) in a large sample of youth with ASD, aged 6 years, 11 months to 15 years, 4 months. Program delivery, both through a tertiary hospital and via community clinics, yielded significant improvements in anxiety following treatment, at levels commensurate with those obtained from controlled research-based studies. An attempt to uncover individual predictors of treatment response did not yield any significant results, suggesting that the program may be widely applicable across the age- and ability-range examined.

The current research builds upon the evidence base supporting the use of the FYF program for youth with ASD and anxiety, and brings the evidence to the necessary next stage in the science-to-service pipeline [47] by demonstrating the program’s effectiveness in community settings. Although implementation processes were not examined systematically, this study lays the groundwork for understanding the context in which this program can be successfully delivered. Next steps include comprehensive evaluation of barriers and facilitators to widespread implementation and uptake. This will inform practice and policy decisions regarding provision of care to individuals with ASD who have co-occurring anxiety that can significantly interfere with everyday function and quality of life. Future clinical and research efforts should continue to build capacity for community-based mental health services for children with ASD with an emphasis on interdisciplinary collaboration. Strengthening relationships between mental health and other interdisciplinary clinicians allows different professionals to bring unique perspectives that can increase access to a range of evidence-based treatments for children and youth with ASD.

## Data Availability

Data set can be made available.

## Appendix A

**Group Questionnaires-Parent and Child versions**

The following four quantitative parent ratings were used in current analyses (scale of 0–8 or 0–5): (1) effective: mean of scores on two items “How effective do you feel at managing your child`s feelings of anxiety?` and “how confident do you feel managing your child`s feelings of anxiety?” (2) Avoidance: “How often does your child avoid situations because of his/her anxiety?”, (3) interference: mean of four items: “In general, how much do you feel that your child`s anxiety is interfering with his/her daily functioning (e.g., stopped him/her from doing things that he/she would like to do, or prevents him/her from participating in activities)?”, “how much do you feel that your child`s anxiety is interfering with his/her functioning within the *school* environment?”, “… within the *home* environment?”, and “… within the *community*?”, and (4) family impact: “how much do you feel that your child`s anxiety is impacting on other family members (e.g., parents, siblings)?”. The following two quantitative child self- ratings were also used: (1) amount of worry: mean of three items, “how much do you worry?”, “how much would your parents say you worry?”, and “do you think you worry more than other kids?”, and (2) Interference/distress: mean of two items, “how much does your worrying get in the way of you doing other things (e.g., having fun, doing school work, falling asleep at night)?” and “how much do your worries bother you?).”

## Abbreviations

AN(C)OVA: Analysis of (Co-) Variance
ASD: Autism Spectrum Disorder
BASC-2: Behavior Assessment System for Children, Second Edition
BSI: Behavioral Symptoms Index
CBT: Cognitive Behavioral Therapy
DSM: Diagnostic and Statistical Manual of Mental Disorders
FSIQ: Full Scale Intelligence Quotient
FYF: Facing Your Fears
ICD: International Statistical Classification of Diseases
RCT: Randomized control trial
SCARED: Screen for Child Anxiety Related Emotional Disorders
SCAS: Spence Children’s Anxiety Scale
SCQ: Social Communication Questionnaire
SRS: Social Responsiveness Scale
TAU: Treatment as usual
WASI: Wechsler Abbreviated Scale of Intelligence
